# Exploratory analysis of potential association between oral *Haemophilus* and sleep disturbances in major depressive disorder patients

**DOI:** 10.3389/fcimb.2025.1617553

**Published:** 2025-07-11

**Authors:** Yachen Shi, En Zhao, Weigang Gong, Qianqian Gao, Yang Li, Guangjun Xi, Yan Han, Hui Weng, Feng Wang, Feng Geng, Gaojia Zhang

**Affiliations:** ^1^ Department of Neurology, the Affiliated Wuxi People’s Hospital of Nanjing Medical University, Wuxi People’s Hospital, Wuxi Medical Center, Nanjing Medical University, Wuxi, China; ^2^ Department of Interventional Neurology, the Affiliated Wuxi People’s Hospital of Nanjing Medical University, Wuxi People’s Hospital, Wuxi Medical Center, Nanjing Medical University, Wuxi, China; ^3^ Department of Gastroenterology, Xishan People’s Hospital of Wuxi City, Wuxi, China; ^4^ Department of Neurology, Qilu Hospital, Cheeloo College of Medicine, Shandong University, Jinan, China; ^5^ Department of Radiology, the Affiliated Wuxi People’s Hospital of Nanjing Medical University, Wuxi People’s Hospital, Wuxi Medical Center, Nanjing Medical University, Wuxi, China; ^6^ Department of Psychology and Sleep Medicine, The Second Hospital of Anhui Medical University, Hefei, China; ^7^ Center for Scientific Research and Experiment, the Second Affiliated Hospital of Anhui Medical University, Hefei, China

**Keywords:** sleep disturbances, major depressive disorder, oral microbiota, haemophilus, glial fibrillary acidic protein

## Abstract

**Background:**

The current study aimed to explore the specific oral microbiota profiles in major depressive disorder (MDD) patients with sleep disturbances, and to evaluate the potential mechanisms by which oral microbiota may be implicated in MDD.

**Method:**

Thirty-eight MDD patients experiencing sleep disturbances and thirty healthy controls (HCs) were included. All MDD patients underwent a 14-day antidepressive treatment regimen. Neuropsychological assessments were conducted, and 16S rRNA sequencing was used to determine the abundance of oral bacteria.

**Results:**

Oral genera *Solobacterium*, *Granulicatella*, *Campylobacter*, and *Haemophilus* showed significant changes in their relative abundances between the MDD and HC groups. Significant correlations were found between the abundance of *Haemophilus* and Pittsburgh Sleep Quality Index (PSQI) and 24-item Hamilton Depression Scale (HAMD-24) scores in MDD patients with sleep disturbances. In MDD patients, lower relative abundances of oral *Haemophilus* prior to treatment were negatively correlated with the changed rates of PSQI and HAMD-24 scores after antidepressive treatment. The glial fibrillary acidic protein as the mediator, affected the relationship between the relative abundance of oral *Haemophilus* and sleep disturbances in MDD patients.

**Conclusion:**

Oral *Haemophilus* dysbiosis may drive sleep disturbances in MDD patients, possibly through its impact on neuroinflammation.

## Introduction

Major depressive disorder (MDD), a leading global disease burden by 2030 (Cui et al., 2024), is characterized by persistent low mood and anhedonia (Anderson et al., 2024). Notably, 60-90% of MDD patients experience sleep disturbances (Malhi and Mann, 2018; Chen RF. et al., 2022), suggesting shared mechanisms involving HPA axis dysfunction and neuroinflammation (Irwin et al., 2016; Szmyd et al., 2021; Bhat, 2024). Furthermore, there exists a bidirectional relationship between gut microbiota dysbiosis and both sleep disturbances and MDD, wherein inflammation and endocrine hormones play pivotal roles (Li et al., 2018; Chen et al., 2024). Microbial metabolism generates an array of neurotransmitters and metabolites, including serotonin, which is intricately linked to the occurrence of rapid eye movement sleep and the manifestation of MDD (Shirolapov et al., 2024). Concurrently, emotional stress and disruptions in the host’s circadian rhythms can induce intestinal dysbiosis and activate intestinal immunity (Yang et al., 2023; Manosso et al., 2024). However, the exact pathophysiology remains unclear, and current treatments [e.g., selective serotonin reuptake inhibitors (SSRIs), serotonin-norepinephrine reuptake inhibitors (SNRIs)] lack reliable efficacy predictors or biomarkers for MDD with sleep disturbances (Wichniak et al., 2017).

The oral microbiome influences brain disorders via neuroinflammation, neuroendocrine regulation, and central nervous system signaling (Bowland and Weyrich, 2022; Zhang et al., 2025). Specific oral bacteria (*Spirochaetaceae*, *Actinomyces*, *Treponema*, *Fusobacterium*) correlate with depressive symptoms through cortisol and CRP modulation (Simpson et al., 2020). Depression-like mice show oral dysbiosis (increased *Pseudomonas*/Pasteurellaceae, decreased *Streptococcus*) linked to blood-brain barrier disruption (Lou et al., 2024). Furthermore, sleep apnea (OSA) patients demonstrate distinct oral microbiota and metabolic pathways, including periodontitis-related species (Bianchi et al., 2023; Gao et al., 2023; Zhang et al., 2023; Ye et al., 2024). However, no studies have examined oral microbiota’s role in sleep disturbances specific to MDD.

The present study aimed to pinpoint the specific oral microbiota in MDD patients with concurrent sleep disturbances, which differ from those present in a healthy condition. Additionally, we further evaluated the association between these specific oral bacteria and various factors, including the severity of sleep disturbances and depression, neuroinflammation marker levels, and the effectiveness of antidepressant therapy.

## Materials and methods

### Participants

There were 68 participants were included in the present study. Thirty-eight MDD patients with sleep disturbances and 30 healthy controls (HCs) were recruited from the Second Affiliated Hospital at Anhui Medical University. All MDD patients met the Diagnostic and Statistical Manual of Mental Disorders, Fifth Edition (DSM-V) criteria (Uher et al., 2014).

The inclusion criteria for patients with MDD encompassed: (1) those experiencing their first episode, whether as outpatients or inpatients; (2) patients who were either naive to pharmacological treatment or had discontinued all antidepressive treatments for a period exceeding four weeks prior to the study’s commencement; (3) patients exhibiting a definitive sleep disorder, as confirmed through sleep assessments; and (4) individuals without a familial history of psychosis. Meanwhile, HCs had no history of DSM-V Axis I disorders, mental health issues, or significant physical ailments, and demonstrated good sleep quality. Furthermore, participants were excluded if they met any of the following criteria: (1) the presence of organic lesions within the central nervous system or neurodegenerative disorders, such as stroke or Parkinson’s disease; (2) secondary mental disorders arising from severe physical conditions; (3) history of alcohol or drug abuse and dependence; (4) significant physical ailments, encompassing endocrine disorders, autoimmune conditions, or impaired liver or kidney function; (4) any form of neoplasia or cerebral trauma; (5) pregnant and breastfeeding women; or (6) history of OSA.

The ethical approval was obtained from the Ethics Committee of the Second Hospital of Anhui Medical University (approval number: SL-YX2024-022). All participants or their legal guardians provided informed consent.

### Follow-up study

All patients diagnosed with MDD underwent a 14-day antidepressive treatment. During this period, only one type of SSRIs or SNRIs was administered as the primary antidepressant. Because of the primary objective of this study was not to assess the efficacy of antidepressive treatment, the study was designed as an observational follow-up rather than a randomized controlled trial.

### Neuropsychological assessments

The interviews of each participant were completed by a trained psychiatrist. Neuropsychological assessments were conducted for HCs at baseline, as well as for patients with MDD both prior to and following treatment.

In the present study, Pittsburgh Sleep Quality Index (PSQI) (Buysse et al., 1989) was performed to measures sleep quality over the previous month. The questionnaire evaluates seven clinically derived domains of sleep quality, sleep onset latency, sleep duration, sleep efficiency, sleep difficulty, sleep medication, and daytime dysfunction. These domains are collectively scored to determine a single factor representing global sleep quality, with a global score exceeding 11 serving as an indicative marker of significant sleep disturbances.

Furthermore, the 24-item Hamilton Depression Scale (HAMD-24) (Hamilton, 1960) and Self-Rating Depression Scale (SDS) (Zung, 1965) were used to evaluate the depressive symptoms. Meanwhile, each MDD patient also received an individual sleep disturbance score, which was computed by summing the scores of items 4, 5, and 6 from the HAMD-24 (Shi et al., 2020a; Shi et al., 2021a; Shi et al., 2021b).

### Collection of oral samples and 16S rRNA gene sequencing

In the current study, oral biospecimens were collected noninvasively from the tongue dorsum using a sterile cotton swab, placed them in a sterile centrifuge tube with 1.5 mL Tris-EDTA buffer solution (Solarbio) and rapidly transported and stored them at -80°C. All participants completed the oral sample collection process after neuropsychological assessment.

Total microbial genomic DNA was extracted using the Bacterial DNA Extraction Mini Kit (Mabio, Guangzhou, China) according to manufacturer’s instructions. The quality and concentration of DNA were determined by 1.0% agarose gel electrophoresis and a NanoDrop^®^ ND-2000 spectrophotometer (Thermo Scientific Inc., USA). The hypervariable region V3-V4 of the bacterial 16S rRNA gene were amplified using specific primers (338F and 806R) by an T100 Thermal Cycler (BIO-RAD, USA). Using the NEXTFLEX Rapid DNA-Seq Kit (Bioo Scientific, USA) to generate the sequencing libraries. Purified amplicons were pooled in equimolar amounts and paired-end sequenced on an Illumina NextSeq 2000 PE300 platform (Illumina, San Diego,USA) according to the standard protocols by Majorbio Bio-Pharm Technology Co. Ltd. (Shanghai, China). Raw FASTQ files were de-multiplexed using an in-house perl script, and then quality-filtered by fastp version 0.19.6 and merged by FLASH version 1.2.11. The optimized sequences were clustered into operational taxonomic units (OTUs) using Usearch 11 with 97% sequence similarity level. The metagenomic function was predicted by PICRUSt2 (Phylogenetic Investigation of Communities by Reconstruction of Unobserved States) based on OTU representative sequences. More details could be found in the **Supplementary Materials**.

### Serum samples collection and detection of serum indicators

Following an overnight fast, peripheral venous blood was collected into a vacutainer tube without anticoagulant. Within 30 minutes after collection, the blood samples were centrifuged at 3500 revolutions per minute at a temperature of 4°C for a duration of 10 minutes, subsequently serum was aspirated and stored at -80°C until needed for analysis. Notably, all MDD patients provided blood samples prior to initiating treatment.

The serum levels of glial fibrillary acidic protein (GFAP) and S100beta protein (S100β) were assayed in triplicate, using the commercial Enzyme-linked Immunosorbent Assay kits (FineTest, Wuhan, China; Catalog Number: EH0410 for GFAP and EH0543 for S100β) in accordance with the manufacturer’s protocols. The protein concentration in each plate was determined based on the standard curves. Both the inter-assay and intra-assay coefficients of variation were less than 5%.

### Statistical analysis

The data were analyzed utilizing SPSS version 22.0 (SPSS, Inc., Chicago, IL, USA) and R software package (version 4.2.1).

Alpha and beta diversity analyses were conducted for the diversity analysis of oral microbiota. In the alpha diversity analysis, Observed species, Chao, and ACE indices were utilized to evaluate the richness of the microbial community, whereas Shannon, Simpson, and Coverage indices were harnessed to assess the diversity of the community. To investigate the variations in the composition of oral microbiota, beta diversity analysis was performed using Partial Least Squares-Discriminant Analysis (PLS-DA). Meanwhile, the microbial dysbiosis index (MDI) acted as an indicator to assess the extent of microbial imbalance within the study population, with a higher MDI value signifying a more pronounced degree of bacterial disturbance (Gunathilake et al., 2020). It was calculated using the formula MDI=log_10_[(total abundance in genera increased in disease group)/(total abundance in genera decreased in disease group)]. Furthermore, MetagenomeSeq analysis was conducted to compare the relative abundances of oral microbiota between the MDD and HC groups (Barot et al., 2024; He et al., 2024).

To ascertain the normal distribution of the data, the Kolmogorov-Smirnov test was performed. For categorical variables, a chi-squared test was employed for analysis. As for continuous variables, the independent-samples t test was utilized when the data were normally distributed; otherwise, the Mann-Whitney U test was applied. The paired t-test was employed to compare the changes in variables before and after treatment. In MDD patients, partial correlation analysis was conducted to ascertain the associations between the two variables, adjusting for factors such as age, sex, years of education, duration of the disease, and body mass index (BMI). Additionally, mediation analysis in MDD patients, controlling for age, sex, education years, duration of the disease, and BMI, was conducted to determine whether oral bacteria mediated the relationship between neuropsychological assessments and serum molecular indicators, based on a standard three-variable mediation model (Baron and Kenny, 1986; He et al., 2019). A detailed description of the method can be seen in **Supplementary Materials**. Receiver operating characteristic (ROC) curves were utilized to calculate the area under the curve (AUC), thereby assessing the diagnostic accuracy of oral bacteria in identifying MDD. The Youden index (Youden, 1950) was used to assess optimal values of sensitivity and specificity. Notable, changed rate of scores=(before treatment score - after treatment score)/before treatment score. Statistical significance was established at a p-value of less than 0.05 (two-tailed).

## Results

### Characteristics of participants

The demographic and clinical characteristics of the participants in the two groups are succinctly summarized in **Table 1**. No significant differences were observed between the two groups in terms of age, sex, education years, and body mass index. When compared to HCs, patients with MDD exhibited significantly elevated scores on the PSQI, HAMD-24, SDS, as well as increased serum levels of GFAP and S100β (**Table 1**). Additionally, there were significant differences in seven factor scores of the PSQI scale and the somnipathy factor scores of HAMD-24 scale between the two groups, as highlighted in **Table 1**.

**Table 1:** Clinical characteristics of all participants.

	MDD (n = 38)	HCs (n = 30)	P-value
Age	26.58 ± 8.69	25.50 ± 3.78	0.319^a^
Sex (female/male)	29/9	19/11	0.243^b^
Education years	11.87 ± 2.72	13.47 ± 3.90	0.106^a^
Duration of the disease (month)	24.08 ± 27.98	–	–
BMI	22.49 ± 4.36	21.89 ± 4.23	0.569^c^
Before treatment			
HAMD-24 scores	27.16 ± 5.38	1.90 ± 1.45	< 0.001^a^
Somnipathy factor scores	4.66 ± 1.12	0.53 ± 0.78	< 0.001^a^
SDS scores	71.87 ± 10.43	38.23 ± 10.20	< 0.001^c^
PSQI global scores	15.26 ± 2.08	4.23 ± 1.52	< 0.001^a^
Sleeping quality	2.66 ± 0.48	0.87 ± 0.43	< 0.001^a^
Sleep onset latency	2.21 ± 0.66	0.87 ± 0.78	< 0.001^a^
Sleep duration	2.03 ± 0.75	0.90 ± 0.55	< 0.001^a^
Sleep efficiency	2.00 ± 0.84	0.23 ± 0.50	< 0.001^a^
Sleep difficulty	1.74 ± 0.69	0.80 ± 0.41	< 0.001^a^
Sleep medication	2.61 ± 0.64	0.03 ± 0.18	< 0.001^a^
Daytime dysfuction	2.03 ± 0.68	0.53 ± 0.57	< 0.001^a^
Serum levels of GFAP	0.21 ± 0.07	–	–
Serum levels of S100β	88.32 ± 21.88	–	–
After treatment			
HAMD-24 scores	9.68 ± 2.72	–	< 0.001^d^
Somnipathy factor scores	2.18 ± 0.93	–	< 0.001^d^
SDS scores	57.82 ± 9.85	–	< 0.001^d^
PSQI global scores	8.47 ± 2.85	–	< 0.001^d^
Sleeping quality	1.55 ± 0.83	–	< 0.001^d^
Sleep onset latency	1.39 ± 0.89	–	< 0.001^d^
Sleep duration	0.97 ± 0.94	–	< 0.001^d^
Sleep efficiency	0.82 ± 0.87	–	< 0.001^d^
Sleep difficulty	1.16 ± 0.86	–	< 0.001^d^
Sleep medication	1.16 ± 1.15	–	< 0.001^d^
Daytime dysfuction	1.42 ± 0.60	–	< 0.001^d^
SSRIs or SNRIs, N%	38 (100.00%)	–	–
Escitalopram	13 (34.21%)	–	–
Sertraline	9 (23.68%)	–	–
Duloxetine	8 (21.05%)	–	–
Fluoxetine	8 (21.05%)	–	–

MDD: major depressive disorder; HC: healthy control; BMI: body mass index; HAMD-24: 24-item Hamilton Depression Scale; SDS: Self-Rating Depression Scale; PSQI: Pittsburgh Sleep Quality Index; GFAP: glial fibrillary acidic protein; S100β: S100beta protein; SSRIs, selective serotonin reuptake inhibitors; SNRIs, serotonin-norepinephrine reuptake inhibitors.

a P-values were obtained by Mann-Whitney U test.

b P-values were obtained by Chi-square test.

c P-values were obtained by Independent-Samples T test.

d P-values were obtained by paired sample t test between before and after treatment in MDD patients.

Following a 14-day antidepressive intervention, MDD patients displayed significant reductions in their PSQI, HAMD-24, and SDS scores when compared to their pretreatment assessment scores (**Table 1**). Additionally, there were notable decreases in seven factor scores of the PSQI scale and the somnipathy factor scores of the HAMD-24 scale (**Table 1**).

### Compositional analysis of oral microbiota

A total 4,024,824 sequences (SRA accession number: PRJNA1279610) were obtained from 68 samples using QIIME software (version 1.91; URL link: https://qiime.org/). The operational taxonomic units were assigned based on a threshold of 97% sequence similarity. The MDD group demonstrated a greater abundance of OTUs compared to the HC group, with a count of 1106 versus 1063, respectively, encompassing 750 shared OTUs (**Supplementary Figure S1**). The rarefaction curves for the samples reached a saturation plateau at a sequencing depth of 42,952 reads, indicating that the majority of microbial species were adequately covered by the sequencing depths employed, and that the sample size was appropriately sized (**Supplementary Figure S2**).

### Diversity analysis

In the alpha diversity analysis, six alpha-diversity indices, i.e., Observed species, Chao1, ACE, Shannon, Simpson and Coverage, were analyzed in the present study. The levels of these six indices showed no significant difference between MDD and HC groups (**Supplementary Figure S3A**). Furthermore, in the beta diversity analysis, PLS-DA analysis was conducted for the reduction of the impact of intergroup differences (**Supplementary Figure S3B**).

### MDI analysis

**Figure 1:**
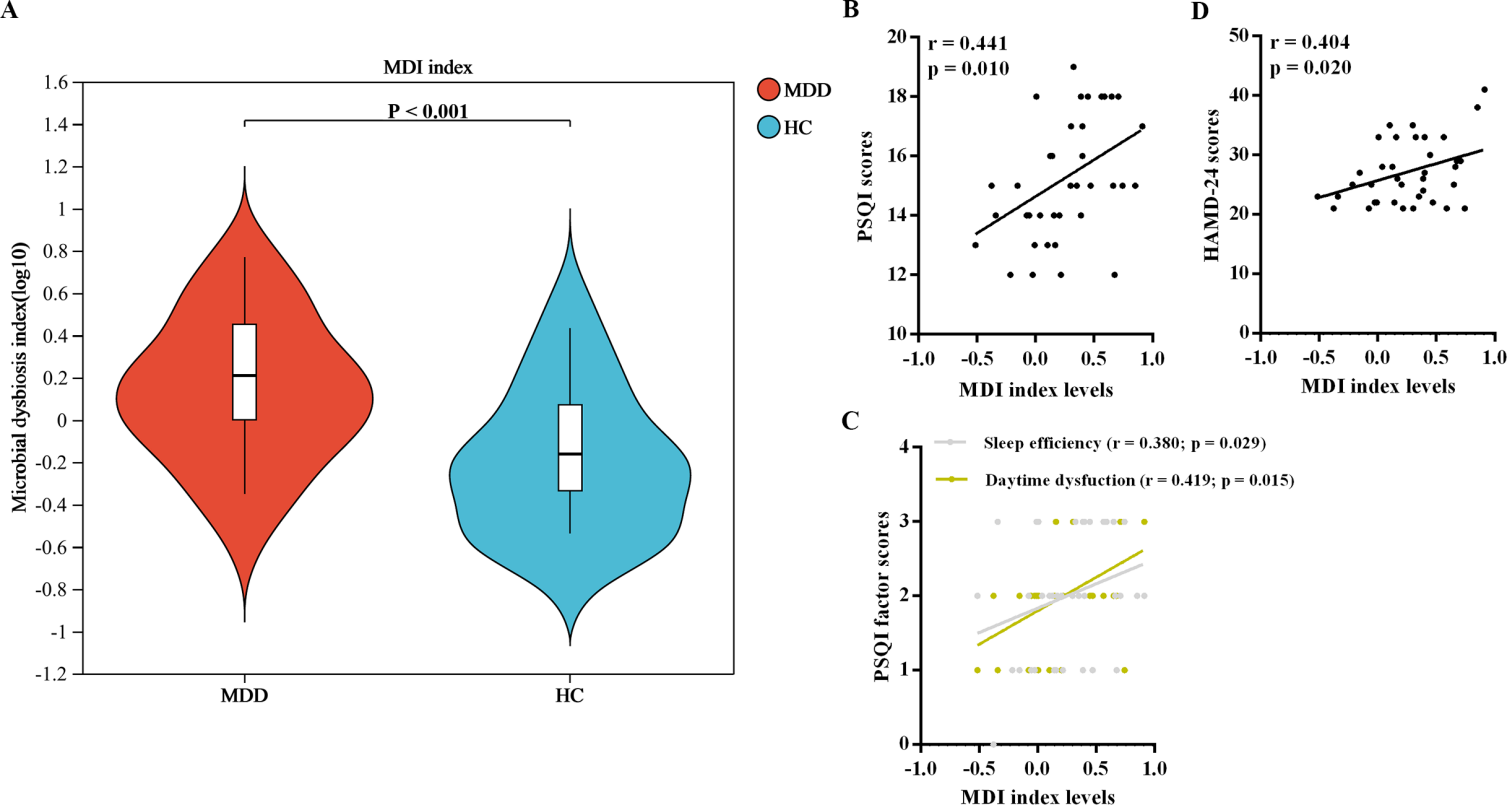
MDI analysis of oral microbiota. **(A)** Comparision of MDI levels between the two groups. **(B)** Correlations between MDI levels and neuropsychological assessments. MDI, microbial dysbiosis index; MDD, major depressive disorder; HC, healthy control.

In the current study, MDD patients exhibited significantly elevated levels of MDI compared to HCs (**Figure 1A**). Furthermore, within the MDD patient group, there were positive correlations observed between MDI levels and scores on the PSQI and HAMD-24 scales, as well as with factor scores pertaining to sleep efficiency and daytime dysfunction, as depicted in **Figures 1B-D**.

### Compositional analysis of oral microbiota at genus level levels between two groups

**Figure 2:**
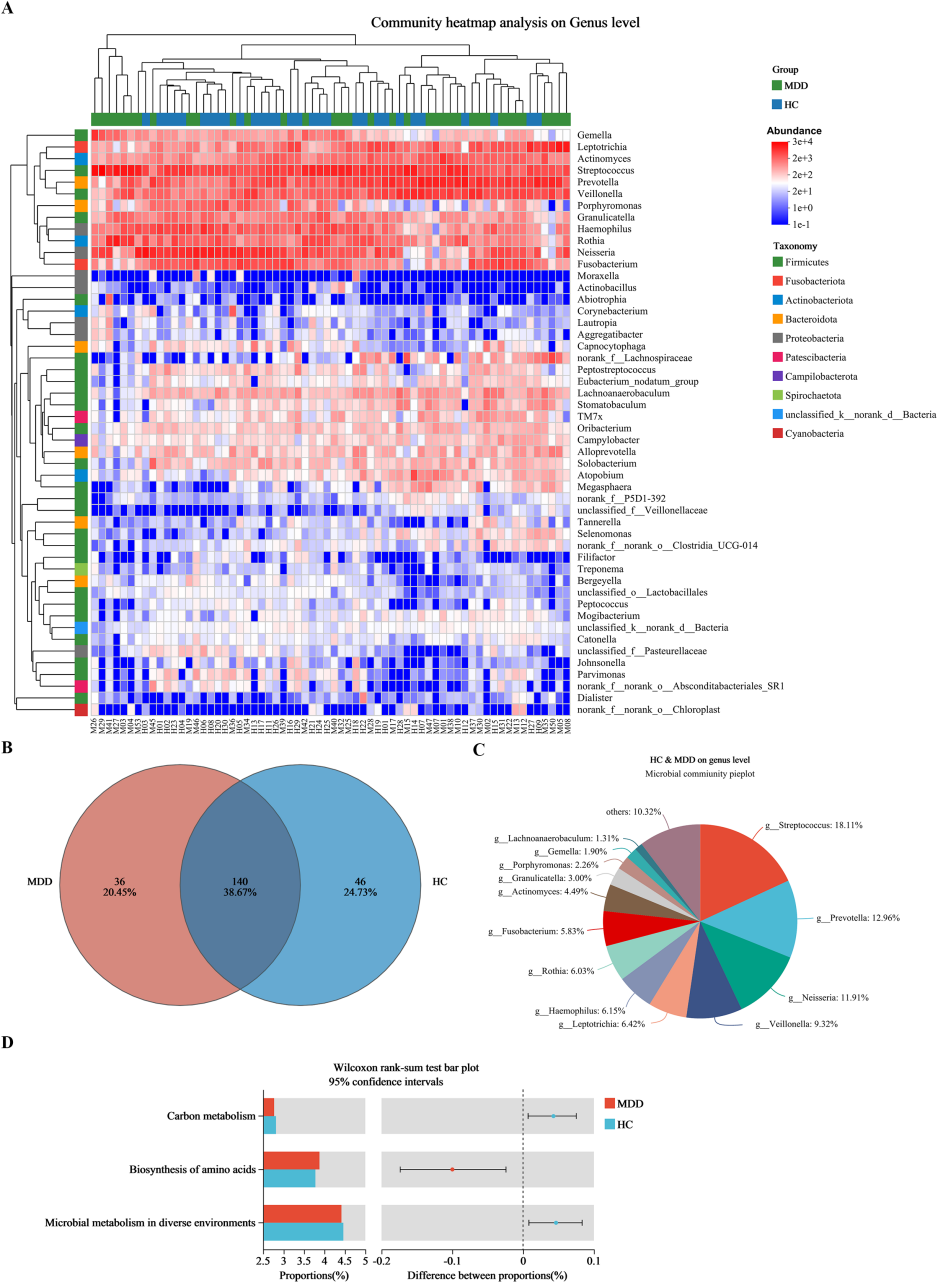
Relative abundances at genus level between MDD and HC groups. **(A)** Heat-map analysis at genus (top 50). Abscissa is the sample and ordinate is the taxa at genus level. The colors in heat-map represent the species abundance. **(B)** The distribution of genus level. **(C)** Composition of the overlapping bacterial genera. **(D)** Predicted functional pathways with significant differences. MDD (M), major depressive disorder; HC (H), healthy control.

**Figure 2A** illustrates the top 50 most prevalent bacterial genera, characterized by their highest relative abundances in both groups. These genera belong to ten different phyla, including *Firmicutes*, *Fusobacteriota*, *Actinobacteriota*, *Bacteroidota*, *Proteobacteria*, *Cyanobacteria*, *etc.* The MDD patients displayed 36 exclusive genera, while 140 identical genera were identified in both the MDD and HC groups (**Figure 2B**). Among the overlapping bacterial genera shared by the two groups, *Streptococcus*, *Prevotella*, *Neisseria*, *Veillonella*, *Leptotrichia*, and *Haemophilus* exhibited the six highest relative abundances (**Figure 2C**).

In addition, the Mann-Whitney U test revealed significant differences in 30 predicted functional pathways between the two groups (**Supplementary Figure S4**). Among these pathways, “Carbon metabolism”, “Biosynthesis of amino acids”, and “Microbial metabolism in diverse environments” demonstrated the highest relative abundances within the oral microbiota (**Figure 2D**).

**Figure 3:**
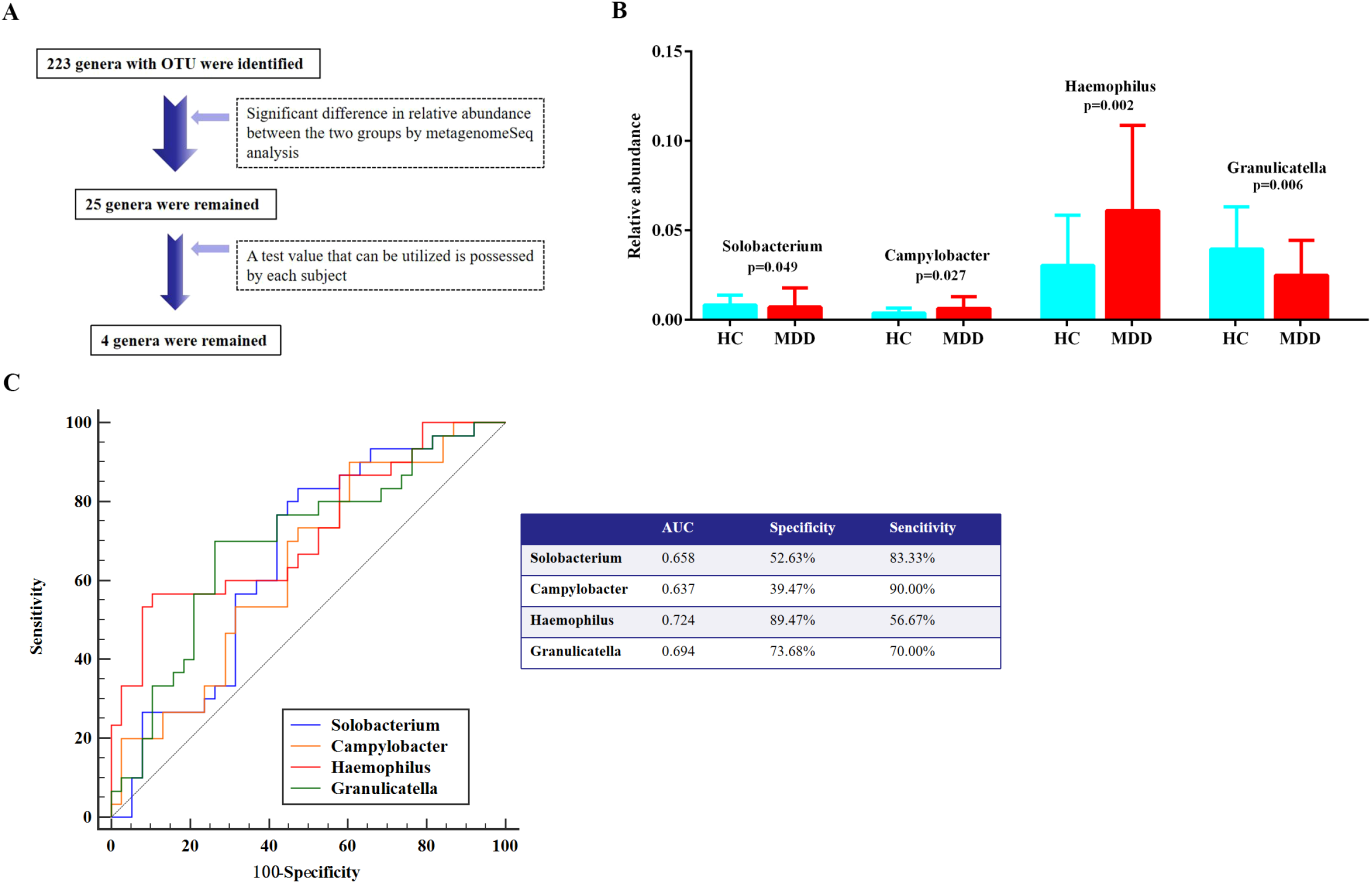
Differentiation of oral microbiota at the genus level in comparative expression analysis between MDD and HC groups. **(A)** Oral microbial screening process. **(B)** Taxa with significant differences at genus level between the MDD and HC groups. **(C)** ROC curve analysis. MDD, major depressive disorder; HC, healthy control; ROC, receiver operating characteristic; AUC, area under the curve.

Furthermore, the MetagenomeSeq analysis represented 25 genera with significant differences in their relative abundances between the two groups (**Figure 3A**). To ensure the precision of the analysis exploring group disparities, four oral bacteria at the genus level were retained, as the test values for these bacteria could reliably be obtained from each subject (**Figure 3A**). Consequently, MDD patients exhibited a significant decline in the relative abundance of *Solobacterium* and *Granulicatella*, whereas *Campylobacter* and *Haemophilus* demonstrated a significant increase, in comparison to the HC group (**Figure 3B**). However, only *Granulicatella* and *Haemophilus* demonstrated statistically significant differences following multiple comparison correction (adjusted p < 0.0125, Bonferroni method).

### ROC curve analysis

To assess the diagnostic efficacy of these four oral bacteria in identifying MDD with sleep disturbances, the ROC curve analysis revealed that *Haemophilus* showed an optimal AUC value of 0.724, accompanied by a sensitivity of 56.67% and a specificity of 89.47% (**Figure 3C**). Additionally, the remaining three oral bacteria demonstrated tolerable diagnostic performance, each possessing an AUC value exceeding 0.6 (**Figure 3C**).

### Association analysis of oral bacteria with environmental factors in MDD patients

**Figure 4:**
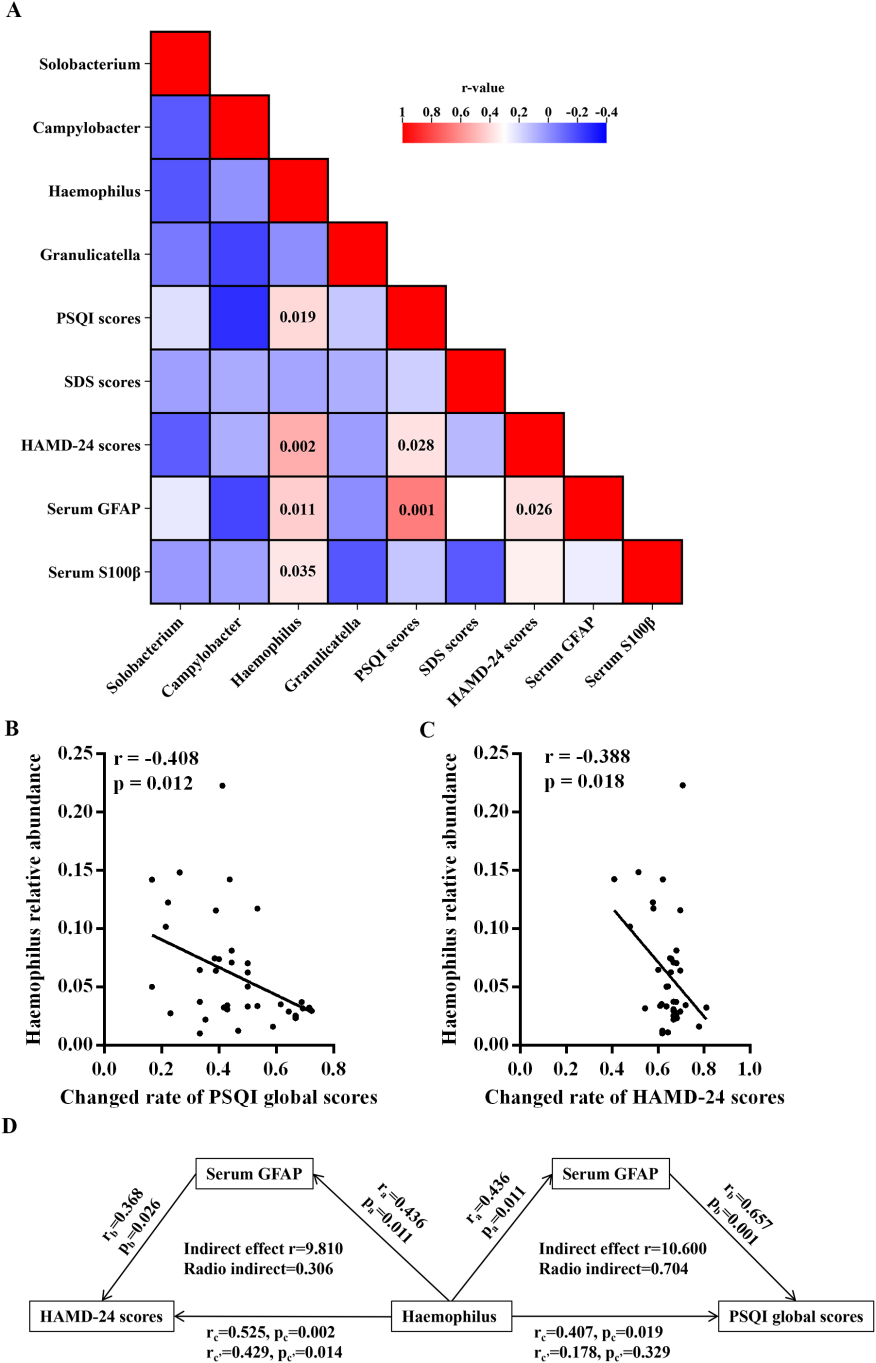
Associations of neuropsychological assessments and serum levels of indicators with oral microbiota at genus level in the MDD group. **(A)** Heatmap shows the correlation coefficient between neuropsychological assessments and serum levels of indicators and oral microbiota at genus level. The numbers in the box are p-values. **(B)** Correlation analyses of the abundance of oral *Haemophilus* prior to treatment with the changed rates of PSQI and HAMD-24 scores after antidepressive treatment. **(C)** Mediation analyses for the association between the relative abundance of oral *Haemophilus* and the assessments of sleep disturbances and depression. MDD, major depressive disorder; HC, healthy control; BMI, body mass index; HAMD-24, 24-item Hamilton Depression Scale; SDS, Self-Rating Depression Scale; PSQI, Pittsburgh Sleep Quality Index; GFAP, glial fibrillary acidic protein; S100β, S100beta protein.

In **Figure 4A**, a correlation matrix was constructed utilizing partial correlation analysis, taking into account environmental factors in MDD patients prior to treatment, while adjusting for variables such as age, sex, years of education, duration of the disease, and BMI. This analysis incorporated four oral microbes at the genus level that exhibited specific alterations in MDD patients. The relative abundance of the genus *Haemophilus* showed significant positive correlations with PSQI and HAMD-24 scores, as well as serum levels of GFAP and S100β (**Figure 4A**). Meanwhile, there were significant correlations between relative abundance of the genus *Haemophilus* and sleeping quality and sleep onset latency factor scores in MDD patients (**Supplementary Table S1**). However, no significant correlations were observed between the other three oral microbes and the environmental factors in MDD patients.

After undergoing a 14-day antidepressive treatment, MDD patients exhibited significant reductions in PSQI, HAMD-24, and SDS scores compared to their pretreatment levels (**Table 1** and **Supplementary Figure S5**). Additionally, correlation analyses were performed for the environmental factors in MDD patients after treatment, with controlling age, sex, years of education, duration of the disease, BMI, and antidepressant medicines. In the MDD group, there were significant negative correlations between the relative abundance of the genus *Haemophilus* and the changed rates of PSQI and HAMD-24 scores (**Figures 4B, C**).

Further mediation analyses conducted in MDD patients revealed that environmental factors played a significant role in affecting the relationship between the relative abundance of oral *Haemophilus* and the severity of sleep disturbances and depression. These analyses accounted for covariates such as age, sex, years of education, duration of the disease, and BMI. Additionally, the serum levels of GFAP were found to influence the relative abundance of *Haemophilus* genera in relation to PSQI and HAMD-24 scores (**Figure 4D**). Notably, serum GFAP exhibited a direct mediating effect on the association between the relative abundance of *Haemophilus* and PSQI scores, as depicted in **Figure 4D**.

### Sample-size estimation

The sample size calculation was performed using an online calculator available at https://sample-size.net/. Based on the relative abundance of *Haemophilus*, with parameters set at α = 0.05 and β = 0.2, the analysis indicated that 27 subjects per group would achieve a statistical power of 0.8082. Consequently, the current sample sizes of 38 and 30 subjects in the respective groups are adequate, as they provide a power value exceeding the conventional threshold of 0.8. Nevertheless, a larger sample size remains necessary to validate the current findings and improve their reliability.

## Discussion

The main findings of this study are summarized below. (1) MDD patients showed significantly higher MDI levels than HCs, and these levels correlated strongly with worse sleep quality and more severe depressive symptoms. (2) At the genus level, oral *Granulicatella* and *Haemophilus* demonstrated significant alterations in their relative abundances between MDD patients and HCs. Notably, *Haemophilus* showed the highest diagnostic accuracy for identifying MDD patients who experience sleep disturbances. (3) In MDD patients, higher abundance of oral *Haemophilus* were associated with poorer sleep quality, more severe depressive states, and increased levels of neuroinflammation. (4) MDD patients who exhibited lower relative abundances of oral *Haemophilus* prior to treatment were more likely to obtain significant improvements in sleep disturbances and depressive symptoms after antidepressant treatment. (5) GFAP-mediated neuroinflammation might be a key mediator in the relationship between Haemophilus abundance and sleep disturbance severity. Therefore, *Haemophilus*-driven oral dysbiosis may significantly contribute to the onset and worsening of sleep disturbances in MDD patients through its neuroinflammatory effects. These findings suggest potential clinical strategies for individualized treatment, including targeted *Haemophilus* antimicrobial or probiotic interventions, which may help alleviate depressive and sleep disorder symptoms while enhancing antidepressant treatment efficacy.

In the current study, a distinct difference in oral microbiota was observed between the two groups, with MDD patients experiencing sleep disturbances demonstrating elevated MDI levels, indicative of more pronounced oral dysbiosis (Wei et al., 2021). Additionally, significant correlations were found between MDI levels and both sleep and depressive assessments, further suggesting that these alterations in oral microbiota expression may may influence sleep disturbances and depressive symptoms. At the genus level, *Solobacterium*, *Granulicatella*, and *Campylobacter* showed marked abundance differences in MDD patients versus HCs, although results of *Solobacterium* and *Campylobacter* could not meet multiple comparison correction. Previous studies have linked these bacteria (*Solobacterium*, *Granulicatella*, *Campylobacter*) to sleep disorders. An increase in the relative abundance of the genus *Solobacterium* was observed in children who exhibit mouth breathing, potentially suggesting a correlation with pediatric OSA (Marincak Vrankova et al., 2024). Concurrently, *Peizeng Jia et al.* discovered that individuals suffering from OSA syndrome exhibited a significantly decreased relative abundance of salivary *Granulicatella* compared to HCs (Jia et al., 2020). Furthermore, a prior study has suggested that individuals with MDD and OSA exhibited an increased abundance of *Campylobacter*, which positively correlated with elevated plasma interleukin-6 levels (Ye et al., 2024). This finding supported the hypothesis that an inflammatory response serves as a potential underlying mechanism linking oral microbial dysbiosis to MDD patients experiencing sleep disturbances. Given the similarity between these prior findings and our current results, our findings possess sufficient credibility. However, in the current study, these three oral bacteria did not demonstrate a strong association with sleep quality or depressive symptoms. This observation may be attributed to the limited sample size or differences in study populations, which could have reduced the statistical power to detect significant relationships. Nevertheless, given their potential clinical relevance, these microbial taxa remain important candidates for future investigation.

In addition, numerous prior studies conducted on diverse cohorts with OSA have reported a significant increase in the abundance of oral *Haemophilus*, potentially implicating them in autoimmune processes (Johnston et al., 2018; Chen X. et al., 2022; Zhang et al., 2023; Zhu and Teng, 2024). Our study provides the first evidence of *Haemophilus* abundance variations specifically in MDD-associated sleep disturbances, which differ from OSA patterns. When compared to HCs, MDD patients with sleep disturbances exhibited a notably heightened relative abundance of oral *Haemophilus*. This oral microorganism holds promise as a potential biomarker for distinguishing individuals with sleep disturbances from those in the HC group. Furthermore, in MDD patients with sleep disturbances, we observed a positive correlation between the relative abundance of oral *Haemophilus* and assessments of both sleep quality and depressive symptoms. While prior research has tentatively indicated that patients with MDD exhibit an elevated expression of oral *Haemophilus* and that antidepressive therapy can diminish this expression (Hoisington et al., 2024), the current findings offer additional insights, suggesting a plausible role for the dysregulation of oral *Haemophilus* in the sleep disturbances experienced by MDD patients. Meanwhile, in the current study, the abundances of oral *Haemophilus* in MDD patients prior to antidepressive treatment exhibited a negative correlation with the rate of change in assessments pertaining to sleep disturbances and depressive symptoms after antidepressant administration. These findings suggest that the elevated expression of oral *Haemophilus* among MDD patients with sleep disturbances may contribute to a reduced efficacy in alleviating these sleep disturbances and depressive symptoms. Previous studies have demonstrated that tricyclic antidepressants (e.g., desipramine) can alter oral microbiota by affecting salivary gland function (Koller et al., 2000a; Koller et al., 2000b). However, the effects of SSRI or SNRI antidepressants on oral microbiota remain unexplored. Further research is needed to determine whether the observed changes in *Haemophilus* abundance following antidepressant treatment result from pharmacological effects or the disease state itself. Therefore, our preliminary study revealed oral *Haemophilus* might be implicated in the onset of sleep disturbances in patients with MDD and affect the prognosis of sleep disturbances following antidepressive treatment. In this preliminary exploratory study, we used 16S rRNA sequencing to characterize oral microbiota composition and identify potential microbial signatures associated with MDD-related sleep disturbances. However, due to the inherent taxonomic resolution limitations of 16S rRNA sequencing, we plan to conduct follow-up investigations using shotgun metagenomics or species-level identification of *Haemophilus* strains and to elucidate their functional pathways more comprehensively.

Peripheral GFAP and S100β are crucial biomarkers indicative of the extent of neuroinflammation and neuronal damage (Steinacker et al., 2021; Zozulya et al., 2021), and they have been established as integral factors in the pathophysiology of MDD (Shi et al., 2020b; Levchuk et al., 2023; Zhao et al., 2024). In this study, significant correlations were identified between the relative abundances of oral *Haemophilus* and serum levels of GFAP and S100β in MDD patients with sleep disturbances, indicating that an increased expression of oral *Haemophilus* may be linked to heightened neuroinflammation. *Alba Troci et al.* also observed an underlying association between oral *Haemophilus*, a Gram-negative, proinflammatory bacterium, and neuroinflammation (Troci et al., 2024), providing additional support for the present finding. Previous studies have demonstrated a correlation between serum levels of GFAP and objective sleep quality in individuals diagnosed with chronic insomnia disorder (Kong et al., 2021), and serum GFAP levels may serve as a biomarker for assessing disease severity in patients with OSA (Guzel and Salış, 2024). In our study, we observed that serum GFAP exerted an indirect mediated influence on the relationship between the relative abundance of *Haemophilus* genus and HAMD-24 scores. Moreover, serum GFAP had a direct mediated effect on the association between the expression of oral *Haemophilus* and PSQI scores. The current data suggest neuroinflammatory involvement in oral *Haemophilus*-associated sleep disturbances. However, whether neuroinflammation serves as the principal mechanism linking oral *Haemophilus* to MDD requires further investigation through fundamental research beyond the scope of this preliminary study.

There were some limitations in the present study. (1) Measurement of the oral microbiota in MDD patients after antidepressive treatment was not completed due to some patients declining to provide oral and blood samples a second time at the end of their treatment. This preliminary study reveals distinct oral microbiota signatures in MDD with sleep disturbances. Future work will employ larger cohorts to track microbiome changes longitudinally before and after antidepressant treatment. (2) Due to the nature of the current study being purely observational, not all MDD patients received the same antidepressant medication during their treatment course. To address this limitation, we will conduct a subsequent randomized controlled trial employing a standardized monotherapy regimen with [e.g., sertraline] to systematically evaluate its effects on oral microbiota composition and clinical outcomes. (3) Our study lacks a comparison group of MDD patients without sleep disturbances. Including such a group would help determine whether the observed bacteria differences are linked to MDD in general or are specific to sleep disturbances in MDD patients. In future research, we plan to validate present findings by comparing depressed patients with and without sleep disorders. (4) This study did not account for potential geographic and dietary influences on oral microbiota composition. Future investigations will account for potential geographic and dietary confounders. We will implement standardized monthly questionnaires to collect these demographic and nutritional data, ensuring comprehensive adjustment in our analytical models. To enhance generalizability, future studies should replicate these findings across diverse racial and ethnic populations. (5) Animal experiments are lacking. It is necessary to conclusively determine whether the artificial introduction of oral *Haemophilus* can expedite the onset of sleep disturbances in animal exhibiting depressive-like behaviors. Furthermore, it is essential to conduct additional fundamental researches to elucidate the underlying mechanism linking oral *Haemophilus* with sleep disturbances in MDD.

## Conclusion

The current study revealed significant dysregulation of oral microbiota in patients with MDD and sleep disturbances compared to HCs. Among the examined oral bacteria, the relative abundance of *Haemophilus* was markedly elevated in MDD patients with sleep disturbances. This increase in oral *Haemophilus* was associated with both the onset and progression of sleep disturbances in MDD patients. Furthermore, GFAP-related neuroinflammation may mediate the relationship between oral *Haemophilus* and sleep disturbances in MDD.

## Data Availability

The original contributions presented in the study are included in the article/**Supplementary Material**. Further inquiries can be directed to the corresponding authors.
