# Supplementary material for: Exploratory analysis of potential association between oral Haemophilus and sleep disturbances in major depressive disorder patients
Source: Front Cell Infect Microbiol. 2025 Jul 11;15:1617553. doi: 10.3389/fcimb.2025.1617553 (PMC12289620; doi:10.3389/fcimb.2025.1617553)
Supplement: Supplementary file 1 [file DataSheet1.pdf]

Supplementary Methods

DNA extraction and PCR amplification

Total microbial genomic DNA was extracted using the Bacterial DNA Extraction Mini Kit (Mabio, Guangzhou, China) according to manufacturer's instructions. The quality and concentration of DNA were determined by 1.0% agarose gel electrophoresis and a NanoDrop® ND-2000 spectrophotometer (Thermo Scientific Inc., USA) and kept at -80 °C prior to further use. The hypervariable region V3-V4 of the bacterial 16S rRNA gene were amplified with primer pairs 338F (5'-ACTCCTACGGGAGGCAGCAG-3') and 806R (5'-GGACTACHVGGGTWTCTAAT-3') by an T100 Thermal Cycler (BIO-RAD, USA). All samples were amplified in triplicate. The PCR product was extracted from 2% agarose gel and purified. Then quantified using Synergy HTX (Biotek, USA). Using the NEXTFLEX Rapid DNA-Seq Kit (Bioo Scientific, USA) to generate the sequencing libraries. Purified amplicons were pooled in equimolar amounts and paired-end sequenced on an Illumina NextSeq 2000 PE300 platform (Illumina, San Diego,USA) according to the standard protocols by Majorbio Bio-Pharm Technology Co. Ltd. (Shanghai, China).

Data processing

Raw FASTQ files were de-multiplexed using an in-house perl script, and then quality-filtered by fastp version 0.19.6 and merged by FLASH version 1.2.11with the following criteria:

(i) the PE300 reads were truncated at any site receiving an average quality score of <20 over a 50 bp sliding window, and the truncated reads shorter than 50 bp were discarded, reads containing ambiguous characters were also discarded; (ii) only overlapping sequences longer than 10 bp were assembled according to their overlapped sequence. The maximum mismatch ratio of overlap region is 0.2. Reads that could not be assembled were discarded; (iii) Samples were distinguished according to the barcode and primers, and the sequence direction was adjusted, exact barcode matching, 2 nucleotide mismatch in primer matching. Then the optimized

sequences were clustered into operational taxonomic units (OTUs) using Usearch 11 with 97% sequence similarity level. The most abundant sequence for each OTU was selected as a representative sequence. The number of 16S rRNA gene sequences from each sample were rarefied to 20,000, which still yielded an average Good's coverage of 99.09% , respectively. The taxonomy of each OTU representative sequence was analyzed by RDP Classifier version 2.13 against the 16S rRNA gene database (e.g. Silva v138) using confidence threshold of 0.7. The metagenomic function was predicted by PICRUSt2 (Phylogenetic Investigation of Communities by Reconstruction of Unobserved States) based on OTU representative sequences.

Mediation analysis

The classic mediation model was selected and then Sobel test was used to confirm the significance of the mediator if the weighted coefficient (a or b) was not significance [1]. Three steps regression models were constructed, as shown below:

$Y = cX + e1$ (1)

$M = aX + e2$ (2)

$Y = c'X + bM + e3$ or $z = \frac{ab}{\sqrt{(b^2 SE_a^2) - (a^2 SE_b^2)}}$ (3)

Where X is the dependent variable (*Haemophilus*), Y is the independent variable (depression or sleep disorder severity), M is the mediator (GFAP), a is the regression coefficient for the relationship between *c* and GFAP strength in distinct region, b is the regression coefficient for the relationship between GFAP in distinct region and HAMD or PSQI score, c is the regression coefficient for the relationship between GFAP on HAMD or PSQI score. z and c' represent the effect of *Haemophilus* on HAMD or PSQI score while controlling for the indirect effect. SEa is the standard error of the relationship between *Haemophilus* and GFAP, and SEb is the standard error of the relationship between GFAP and HAMD or PSQI score. Potential confounding variables, including age, sex, education level, disease duration, and BMI, were included as covariates in all regression analyses to control for their potential

effects in the mediation models.

In this analysis, four conditions for establishing mediation are: 1) c must be significant; 2) a and b are significant; 3) c’ < c (in absolute value, partial mediation) or c’ is insignificant (full mediation); 4) if a or b is insignificant, the Sobel test Z must be significant. If the mediator is existed, we using ratio indirect to present the strength of mediation ((a*b)/c).

Supplementary Table 1. Correlation analyses between the relative abundance of four oral microorganisms and seven factor scores derived from the PSQI scale.

	Solobacterium	Campylobacter	Haemophilus	Granulicatella
Sleeping quality	r = 0.063; p = 0.706	r = 0.033; p = 0.845	r = 0.407; p = 0.011	r = -0.129; p = 0.440

Sleep onset latency	r = -0.260; p = 0.115	r = -0.301; p = 0.066	r = 0.359; p = 0.027	r = 0.180; p = 0.280
Sleep duration	r = 0.014; p = 0.935	r = -0.048; p = 0.774	r = -0.037; p = 0.827	r = 0.378; p = 0.059
Sleep efficiency	r = 0.003; p = 0.985	r = -0.218; p = 0.189	r = 0.264; p = 0.109	r = 0.133; p = 0.426
Sleep difficulty	r = -0.158; p = 0.344	r = -0.046; p = 0.782	r = -0.068; p = 0.685	r = -0.025; p = 0.883
Sleep medication	r = 0.090; p = 0.589	r = -0.021; p = 0.901	r = 0.108; p = 0.517	r = -0.111; p = 0.507
Daytime dysfuction	r = -0.350; p = 0.031	r = 0.171; p = 0.305	r = 0.071; p = 0.672	r = -0.068; p = 0.683
Somnipathy factor scores	r = -0.064; p = 0.704	r = -0.078; p = 0.641	r = 0.285; p = 0.083	r = 0.037; p = 0.827

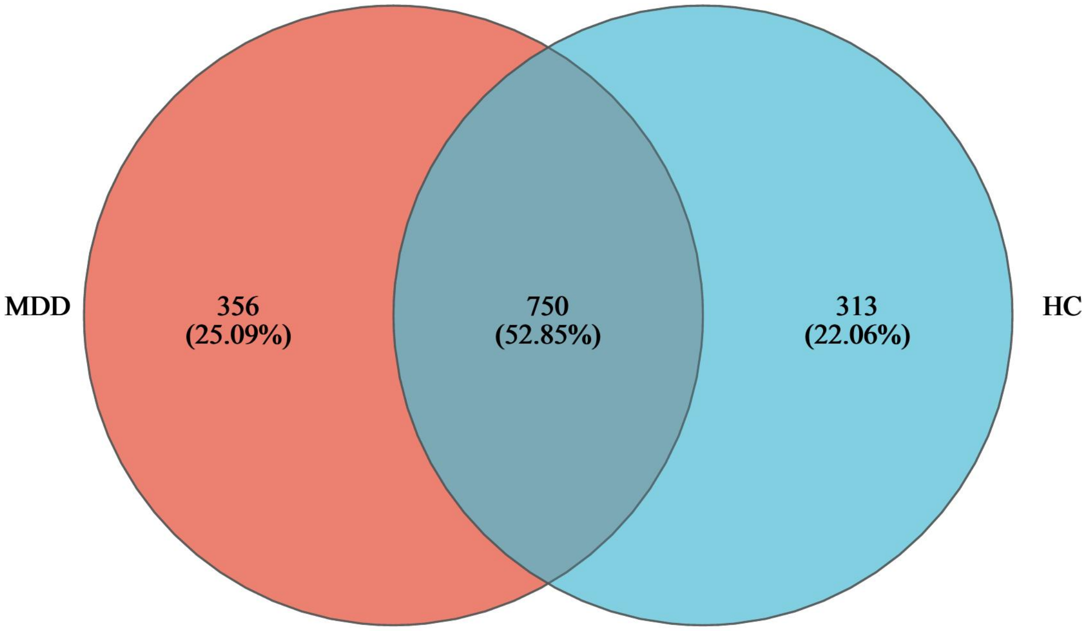

Supplementary Figure 1. The number of OTUs in MDD and HC groups.

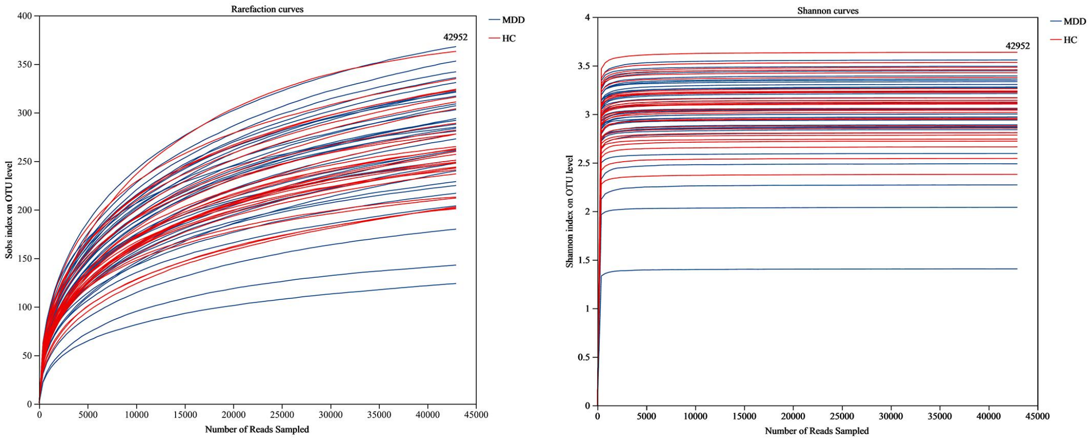

Supplementary Figure 2. The rarefaction curves using α-diversity indices.

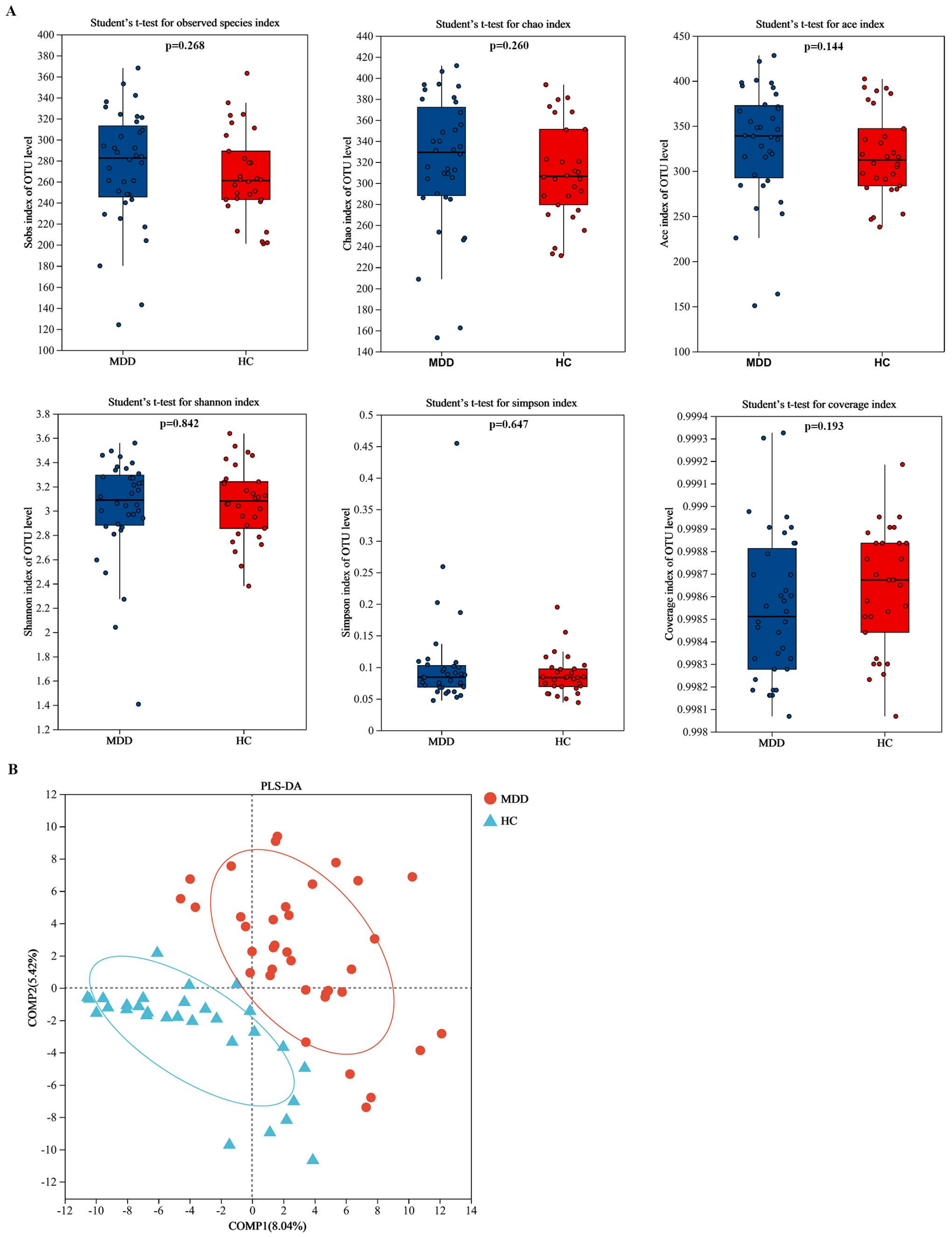

Supplementary Figure 3. Alpha-diversity and Beta-diversity indices for the species in the oral microbiota between MDD and HC groups.

(A) α-diversity indices. (B) β-diversity indices with PLS-DA analysis. The scale stands for the relative distance without practical significance.

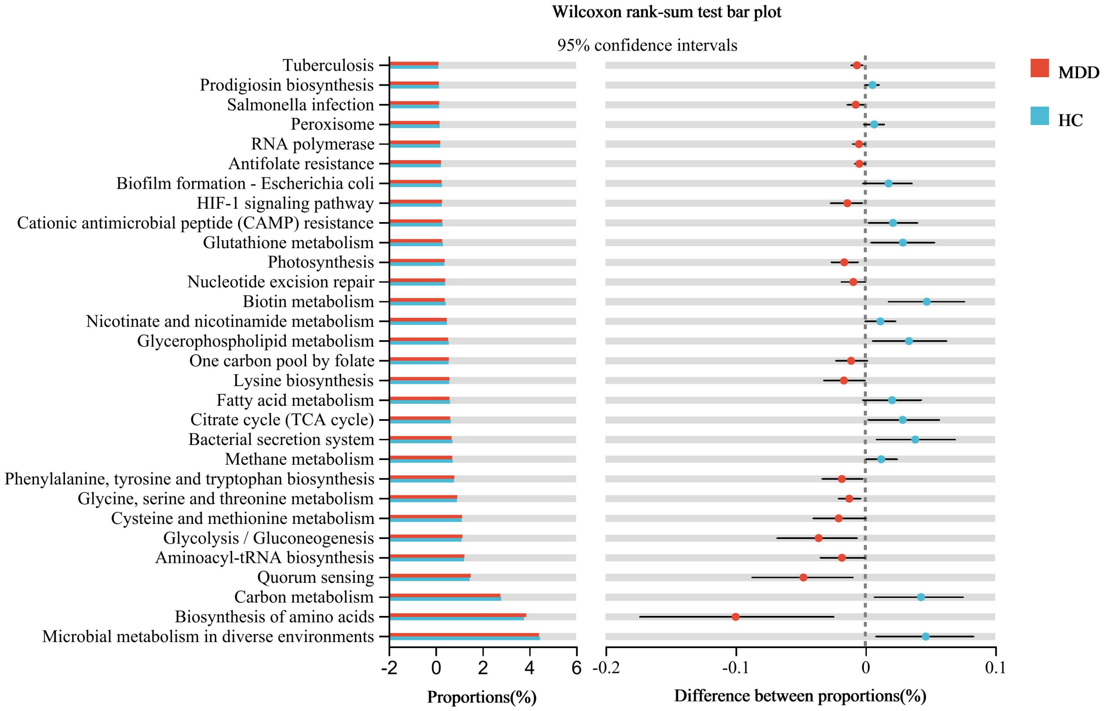

Supplementary Figure 4. Functional predictions of the oral microbiota.

Significant difference in Kyoto Encyclopedia of Genes and Genomes pathways for oral microbiota in MDD and HC groups. The differences in the functional prediction between the two groups were analyzed using Mann-Whitney U test. P-value < 0.05.

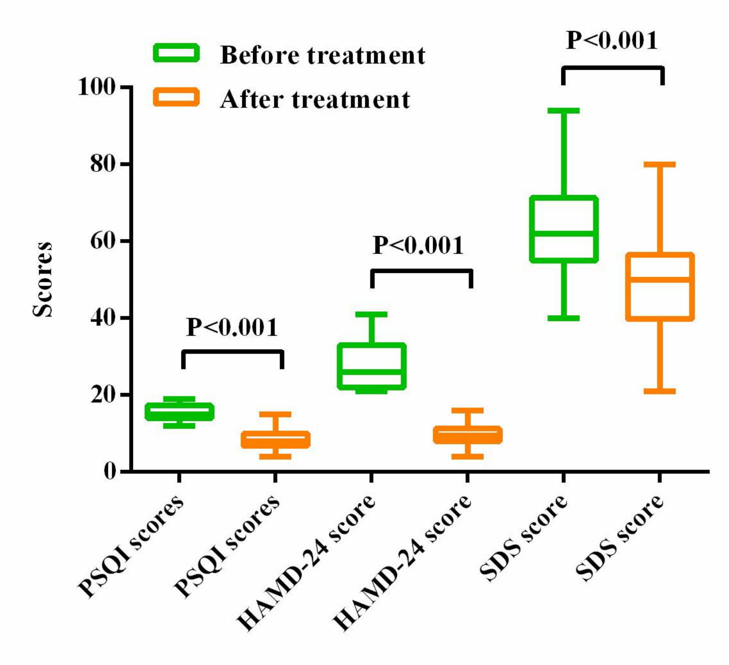

Supplementary Figure 5. Comparison of neuropsychological assessments before and after treatment in MDD patients.

The data were analyzed using paired sample t test.

Reference

1. Baron, R.M. and D.A. Kenny, The moderator-mediator variable distinction in social psychological research: conceptual, strategic, and statistical considerations. J Pers Soc Psychol, 1986. 51(6): p. 1173-82.
